# Machine Learning-Based Identification of Plastic Types Using Handheld Spectrometers

**DOI:** 10.3390/s25123777

**Published:** 2025-06-17

**Authors:** Hedde van Hoorn, Fahimeh Pourmohammadi, Arie-Willem de Leeuw, Amey Vasulkar, Jerry de Vos, Steven van den Berg

**Affiliations:** 1 Photonics Research Group, The Hague University of Applied Sciences, 2628 AL Delft, The Netherlands; 2 AI & Data Science Expert Group, The Hague University of Applied Sciences, 2628 AL Delft, The Netherlands; 3 Plastic Scanner Project, 2611 AN Delft, The Netherlands

**Keywords:** spectroscopy, handheld, machine learning, benchmarking, plastic identification

## Abstract

Plastic waste and pollution is growing rapidly worldwide and most plastics end up in landfill or are incinerated because high-quality recycling is not possible. Plastic-type identification with a low-cost, handheld spectral approach could help in parts of the world where high-end spectral imaging systems on conveyor belts cannot be implemented. Here, we investigate how two fundamentally different handheld infrared spectral devices can identify plastic types by benchmarking the same analysis against a high-resolution bench-top spectral approach. We used the handheld Plastic Scanner, which measures a discrete infrared spectrum using LED illumination at different wavelengths, and the SpectraPod, which has an integrated photonics chip which has varying responsivity in different channels in the near-infrared. We employ machine learning using SVM, XGBoost, Random Forest and Gaussian Naïve Bayes models on a full dataset of plastic samples of PET, HDPE, PVC, LDPE, PP and PS, with samples of varying shape, color and opacity, as measured with three different experimental approaches. The high-resolution spectral approach can obtain an accuracy (mean ± standard deviation) of (0.97 ± 0.01), whereas we obtain (0.93 ± 0.01) for the SpectraPod and (0.70 ± 0.03) for the Plastic Scanner. Differences of reflectance at subsequent wavelengths prove to be the most important features in the plastic-type classification model when using high-resolution spectroscopy, which is not possible with the other two devices. Lower accuracy for the handheld devices is caused by their limitations, as the spectral range of both devices is limited—up to 1600 nm for the SpectraPod, while the Plastic Scanner has limited sensitivity to reflectance at wavelengths of 1100 and 1350 nm, where certain plastic types show characteristic absorbance bands. We suggest that combining selective sensitivity channels (as in the SpectraPod) and illuminating the sample with varying LEDs (as with the Plastic Scanner) could increase the accuracy in plastic-type identification with a handheld device.

## 1. Introduction

Primary plastic production and its ensuing waste has been growing rapidly for many decades and will continue to increase in the foreseeable future [1]. Since 1950, more than 8300 million metric tons (Mt) have been produced, of which 6300 million Mt has resulted in waste while only 12% has been recycled worldwide. Almost 80% of plastic has been accumulated in landfills or the natural environment with detrimental environmental consequences. A wide body of knowledge has been developed in recent years on the ecotoxicological consequences of plastic ingestion and accumulation, while much is not yet known about the exact impact of microplastics. However, it is clear that the flow of plastic waste is ever-growing and that a significant part ends up in the ocean [2].

To decrease plastic waste and increase recycling, it has been suggested that the main focus for this should be low- and middle-income countries where waste management needs most improvement [3]. However, an economic analysis also shows that cost-benefit analysis here does not work in favor of plastic recycling [4]. As such, any solution implemented for recycling in low- and middle-income countries needs to be low-cost. Furthermore, the first step in recycling is sorting, which is often performed by hand. A low-cost, handheld device may aid in identifying plastic types to benefit plastic sorting and recycling in low- and middle-income countries where only a very small fraction of plastics is currently recycled.

From examination of the literature, it is clear that plastic identification can be performed very accurately using near-infrared (NIR) reflection spectroscopy [5,6,7]. In particular, absorbance bands in the short-wave infrared (SWIR) wavelength range (700–2500 nm) are specific to a wide range of plastic types. However, high-end spectroscopy and/or spectral imaging solutions are not feasible as a low-cost solution that can be used in low- and middle-income countries. Controlled lighting conditions and a conveyor for in-line sorting used in high-end recycling plants are technically too complex and the cost of implementing such a system is simply too high. Furthermore, other plastic identification methods exist, but these methods are invasive, more expensive and are research-intensive (and thus, labor-intensive) and thus not relevant options for recycling [8]. Low-cost, handheld NIR devices to assist in sorting may provide a solution for recycling in low- and middle-income countries.

Here, we present a study comparing a high-resolution near-infrared spectrometer (NIRS) to two different low-cost, handheld spectrometers to classify plastic types. One handheld device uses integrated photonics to detect 16 different bands in the SWIR region [9], sold as a device named SpectraPod (SP) by the company MantiSpectra. The other is a handheld device using light-emitting diode (LED)-illumination, produced by the open-source project Plastic Scanner (PScanner) (see www.plasticscanner.com (accessed 8 May 2025)). We measured a wide variety of plastic samples sourced from various locations to ensure thorough testing. To determine the accuracy of classification using all three methods, we used machine learning (ML). We compare different ML approaches, determine their performance, and discuss how well the handheld devices considered perform and seek to explain their performance based on their technical function.

## 2. Materials and Methods

In this research, we combined spectroscopy using different devices with machine learning methods. First, we present our spectroscopic experiments and the plastic samples we measured. Then, we discuss the analytical approaches using machine learning that we applied to the data acquired with the three diferrent devices.

### 2.1. Materials

**Figure 1:**
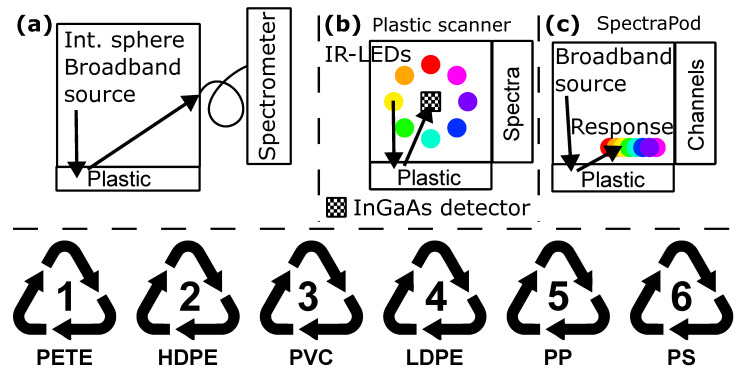
The three different spectroscopic methods compared in this research to identify plastic types 1–6. We measured plastic samples with (**a**) an integrating sphere, broadband source and high-resolution near infrared spectrometer (NIRS) from 1050–1950 nm, (**b**) the discrete illumination-based Plastic Scanner with LEDs ranging from 900 to 1800 nm (PScanner) and (**c**) an integrated photonics approach using the SpectraPod (SP) using broadband light providing spectral responsivity from 850 to 1700 nm. NIRS gives a high-resolution spectrum with 237 datapoints, the Plastic Scanner, a discrete spectrum with 8 points, while the SP gives 16 channels that each have a different responsivity, as given in [9]. Below: Resin identification codes for plastic types used in this research.

In this study, we used three different approaches for reflection spectroscopy of a wide variety of the most common plastic types: (1) polyethylene terephthalate (PET/PETE), (2) high-density polyethylene (HDPE), (3) polyvinylchloride (PVC), (4) low-density polyethylene (LDPE), (5) polypropylene (PP) and (6) polystyrene (PS). These plastic types can be recognized by their resin identification codes, as shown in the bottom of Figure 1. We sourced samples from a wide range of sources, such as medical waste, consumer plastics and packaging material. We collected data on 44 PET, 39 HDPE, 22 PVC, 13 LDPE, 56 PP and 24 PS samples. Each sample was measured multiple times in different orientations, yielding 50–200 scans per sample per plastic type for each device.

### 2.2. Spectroscopy

As depicted in Figure 1a, we measured all samples using a near-infrared spectrometer with 237 wavelength bands (NIRS, Avaspec-NIR256-2.0TEC, Avantes, Apeldoorn, The Netherlands). Wavelength accuracy was ensured with an argon calibration lamp (Avalight-CAL-Ar, Avantes, Apeldoorn, The Netherlands). We used an integrating sphere (AvaSphere-50-LS-HAL-12V, Avantes) to measure the reflectance relative to a diffuse white calibration standard (Zenith SG 3151, Sphere Optics, Herrsching, Germany). We used the built-in broadband halogen lightsource for illumination, also using a dark reference for dark current correction. All measurements were performed at room temperature (21 ± 1) °C in a dark optical laboratory.

We also measured all plastic samples with the Plastic Scanner (PScanner) as depicted in Figure 1b. This is a discrete spectrum illumination-based device with 8 LEDs. By sequential illumination, dark correction and white reference, we were also able to measure a reflection spectrum. This handheld device has a single InGaAs detector with responsivity from 900 to 1700 nm. The entire device is open-source and all information is available online at www.plasticscanner.com (accessed 8 May 2025). As with the NIRS approach, this resulted in a reflectance spectrum, yet this time with 8 wavelengths, as defined by the illumination spectrum of the LEDs. In both the NIRS and PScanner approaches, reflectance was quantified according to the following equation:

(1) $$R=\frac{I-I_{dark}}{I_{ref}-I_{dark}}$$

The third device (see Figure 1c) we used to measure the same set of plastic samples was the SpectraPod (SP, Mantispectra, Eindhoven, The Netherlands) [9]. This device measures the reflectance over multiple wavelengths, and has responsivity curves with spectral bands for its 16 different channels. Therefore, there is no direct comparison possible with the other devices, as this device does not produce reflectance spectra but a combined spectral response over several bands. These bands are in the same wavelength range as for the NIRS and PScanner approaches and, therefore, enable identification of plastic types, as was shown in a previous study [10]. Instead of reflectance datapoints at specific wavelengths, with the SP approach, we train the ML model on the differential response at its channels for the different plastic types.

### 2.3. Machine Learning Analysis

Classification models were developed for all three devices (NIRS, PScanner and SP) separately. We first discuss the feature engineering aspect of our analysis and then elaborate on the procedure for constructing the classification models.

#### 2.3.1. Feature Engineering

Two groups of features were used as input for our classification models. The first group of features was obtained directly from spectroscopy measurement with the reflectance at each available wavelength (or channel) being a separate feature. Hence, this group contained 237, 16 and 8 features for the NIRS, SP and PScanner approaches, respectively. As an additional group of features, we determined the difference between the reflectance at two consecutive wavelengths or channels (the first derivative). For example, the PScanner has one feature as the difference between the reflectance at 1200 nm and 1300 nm. By calculating the differences for all available wavelengths, we constructed 236, 15 and 7 additional features for the three devices. Hence, the total number of features was 473, 31 and 15 for the NIRS, SP and PScanner, respectively. Before applying the classification algorithms, the features were standardized with respect to the average values and standard deviation for all samples in the dataset (e.g., training set) considered for constructing the classification model.

In addition to determining the accuracy of the plastic-type classification models for the three devices, our second aim was to obtain insights about the most relevant features for accurate plastic-type classification. In particular, we calculated which features had the largest impact on the accuracy of the classification model for the NIRS. This enables us to extract insights that potentially can be used for making adjustments to the SP and PScanner to increase their plastic-type classification accuracy. For example, information can be obtained about which combination of LEDs in the Plastic Scanner could be beneficial for accurate plastic-type classification.

Before determining the importance of each feature in the plastic-type classification model, we performed feature selection to ensure collinear features are not present in our entire feature collection for the NIRS. First, we determined the Spearman rank-order correlation ρ for all 473 features. Then, we performed hierarchical clustering using Ward’s linkage on the correlation matrix. The maximal inter-cluster distance was set to 1 and one feature was selected randomly from all distinct clusters. This procedure reduced the number of features to 24. These remaining features were further analyzed for multicollinearity and features with |ρ|> 0.7 were removed. For each group of collinear features, the feature with the smallest average absolute correlation with the other 23 features was included. Finally, we ended up with 20 features for the NIRS.

#### 2.3.2. Classification Models

The plastic types were classified based on features constructed from the reflection spectroscopy data. To obtain unbiased estimates for the generalizability of our models on unseen data, we performed 5-fold nested cross-validation [11]. In brief, this entailed the dataset being split into five distinct parts using stratified sampling where each part contained 20% of the measured samples. One of these parts was selected as the test set and the remaining parts were combined into a single training set. This training dataset was then used to construct a model and the hyperparameter values were tuned using 10-fold inner cross-validation. Finally, we validated this model using the test set. This procedure was repeated five times, with the test dataset being equal to each of the five distinct parts.

A priori, the most suitable algorithm for plastic-type classification was unknown. Therefore, a selection of algorithms were explored to identify the most appropriate classification model. Here, we considered Support Vector Machines (SVM) [12], XGBoost [13], Random Forest [14] and Naïve Bayes [15]. For all the algorithms, models were constructed on the training set using 10-fold (inner) cross-validation and optimizing the hyperparameters (e.g., the maximal depth of a decision tree in the Random Forest algorithm) using grid search. We selected the combination of hyperparameters with the highest average classification accuracy, i.e., the largest fraction of correctly classified samples, for this 10-fold (inner) cross-validation procedure. Then, with a model based on the entire training set from these hyperparameter values, we made predictions on the independent test set. Finally, we compared the predictions to the known classes and determined the accuracy. The classification accuracy, represented as (mean ± standard devation) over the five folds, represents the generalizability of our model.

To obtain more detailed information about the performance of our classification model on unseen data, we also determined the confusion matrix. The confusion matrix consists of two axes. The first axis represents the actual plastic types and the second axis specifies the predictions obtained from the model. Hence, the confusion matrix allows us to evaluate the performance of our classification models across different plastic types and to discuss its strengths and weaknesses in more detail. We determined the confusion matrix for each of the five folds separately. To obtain an overall assessment of the classification models, we present the sum of these five confusion matrices.

After assessing the generalizability of our modeling approach and determining the confusion matrices, we continued by constructing our final model on the entire dataset. First, we optimized the hyperparameters of the algorithms by using 10-fold cross-validation. After constructing our final models on all the available data using the optimal hyperparameter values, we determined the feature importance values using permutations [14]. The feature importance scores were calculated as the percentage decrease in accuracy with respect to the accuracy of the final model. The scores were determined using 10 different random permutations, with the mean and standard deviation of the five most important features being reported.

The analyses were performed in a custom-written Python script (version 3.12.7). The main packages used were scikit-learn, scipy and xgboost.

## 3. Results and Discussion

### 3.1. Plastic-Type Spectroscopy

The number of measurements per class and per spectroscopy method are shown in Table 1. All the different plastic types are well represented for the three devices. The origin of the plastic types varied widely—from packaging material to medical waste—and as such, our samples exhibited wide variation in opacity and color. However, because the materials were sourced from various locations, we could not be sure of the exact coating or other treatment on top of the plastics. This caused some spectra to be flat in the infrared (as observed in the NIRS results) due to an additional layer of material on top of the plastic that caused the characteristic dips in reflection for that type to be missing. From [5,6,7] and our own measurements on pure plastic samples, we understood that specific absorption bands should be present in the reflectance spectrum, so we identified the NIR spectra where these bands were not present. These spectra were classified as unknown in a seperate category, and these samples were also treated as such for the SP and PScanner analysis. In total, we analyzed over 2400 measurements, divided among three devices and distributed over seven classes (six plastic types and one unknown class). This classification was used as the starting point for supervised machine learning.

**Figure 2:**
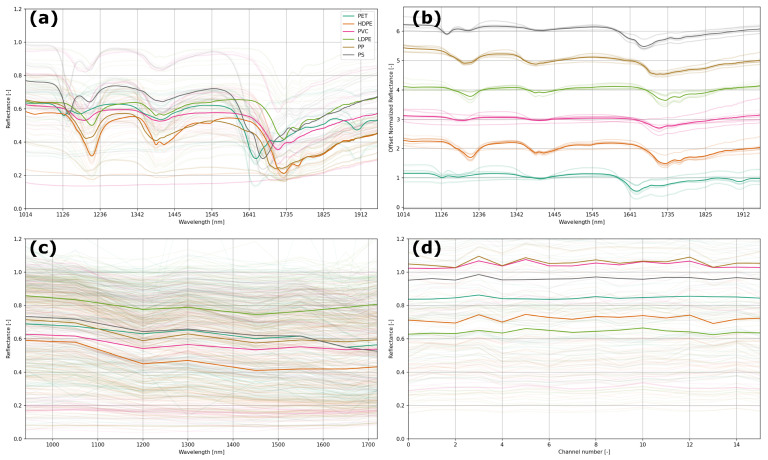
Spectral reflectance measurements obtained with (**a**,**b**) NIR Spectrometer, (**c**) Plastic Scanner and (**d**) SpectraPod (Note: the SpectraPod integrates reflectance over multiple infrared bands as described in [9]). All measured spectra for a variety of plastic samples of different types (e.g., varying colors and opacity) are shown dimly in the background, and the mean of all measurements is given in a solid color corresponding to the legend. (**a**) Displays all NIRS data in one graph, and (**b**) shows the normalized spectra (mean = 1.0) with an offset to more clearly show the spectra.

The experimental results obtained with NIRS and the handheld spectral devices are displayed in Figure 2. We measured a wide variety of plastics of different types, opacities, and colors to ensure a robust approach to classify the plastic types. All measurements were performed on the same overall set of samples (see methods). As input for our further analysis, we used the reflectance as a function of the wavelength for the NIR Spectrometer (NIRS) and Plastic Scanner (PScanner) (see Figure 2a–c), whereas we used the reflectance (as always, relative to a white reference) per channel number for the SpectraPod (SP) (see Figure 2d). In all cases, we used a white reference and a dark measurement to obtain the reflectance relative to a diffuse white reference.

Figure 2a displays all the measurements taken using the NIRS in the background with the mean of all reflectance curves in a solid color, with plastic types depicted according to the legend. Similarly, all data for the PScanner and SP are presented in Figure 2c,d, respectively. Figure 2b shows the offset and normalized reflectance curves (same data as in Figure 2a), which demonstrates clear individual spectral signatures in the infrared curves of different plastics as expected [5,7].

Note that the data of the three devices cannot be compared directly because of the different nature of the data. The spectrometer provides a high spectral resolution, while the PScanner shows much lower spectral resolution and the response is averaged over the LED bandwidth. As the SP integrates reflectance over multiple infrared bands, only a signal for each color pixel is obtained, which cannot be directly correlated to the spectral reflectance of a material. As a consequence, when we use discrete spectroscopic devices, such as the PScanner and SP, it is not possible to see a clear signature in the curves. Measurements with the PScanner (Figure 2c) were performed in the same wavelength range up to approximately 1700 nm, yet a plastic-type dependent signature is hard to distinguish from the curves. Also in the SP measurements (Figure 2d), it is very difficult to identify a clear trend in the pseudo-spectra (’pseudo’ since the channels on the x-axis correspond to sensitivity over several parts of the spectral range from 850 to 1700 nm [9]). Previous research using the SP [10] has shown that it can detect PET, HDPE, LDPE, PP and PS with high accuracy. However, in our dataset, we also included PVC and we measured a very wide collection of plastic samples of different colors and opacities.

While discrete spectroscopic data do not directly provide clear wavelengths at which plastic types can be identified (as could be provided using the spectra in Figure 2a,b), modern machine learning (ML) tools may provide an alternative, robust approach to identifying plastic types with different colors and opacities. We therefore applied various ML approaches to see how well plastic types can be classified using near-infrared reflectance data as obtained with handheld spectroscopic devices compared to a high-resolution spectrometer.

### 3.2. Classification Using Machine Learning

The accuracies of the plastic-type classification models for the three devices are shown in Table 2. We obtain high-precision plastic-type classification (accuracy > 0.9) for the NIRS and SP. For the PScanner, the accuracy of the algorithms is lower. However, the performance of the best plastic-type classification models is still reasonably accurate. For all three devices, the Gaussian Naïve Bayes algorithm is the worst-performing algorithm. The SVM model performs well for all devices, though the Random Forest approach outperforms the other models on the NIRS data. The best-performing models were obtained using sklearn version 1.5.1 and optimizing the hyperparameters C, kernel and gamma, as specified in the module SVC or the hyperparameters max_depth and n_estimators for the module RandomForestClassifier.

Compared to previous research aiming to identify plastic type using a low-cost, hand-held approach, our accuracy is higher compared to truly low-cost approaches, though there are significant differences in the research. Ou et al. [10] showed a high accuracy of 100% using the SP on a fairly homogeneous test set (regarding sample color and transparancy); however, they did not include PVC in the classification. Martinez et al. [16] recently published an article about low-cost recognition of plastic waste with a multi-spectral sensor. They investigated the same plastic types as in our research and managed to achieve accuracies of 0.50–0.70, but it appears the accuracy was low because the wavelength detection range was limited to λ<1000 nm. As they and others have shown, higher accuracy detection is attainable but only with much more expensive sensors. Pakhomova et al. [17] also reported a low-cost approach, but show high cross-correlation for multiple plastic types when classifying, even though they used an NIR spectrometer up to 1700 nm (using a small yet not very low-cost spectrometer). West et al. [18] used a low-cost approach but also only achieved an accuracy of 0.62.

Overall, the very-low-cost result obtained using the PScanner, with an accuracy of 0.70 and the SP attaining 0.93, represents a step forward in low-cost, handheld plastic-type identification. Since both the PScanner and SP do not need a linear InGaAs detector or a diffractive optical element, these approaches are both robust and low-cost.

### 3.3. Cross-Correlation and Feature Importance

**Figure 3:**
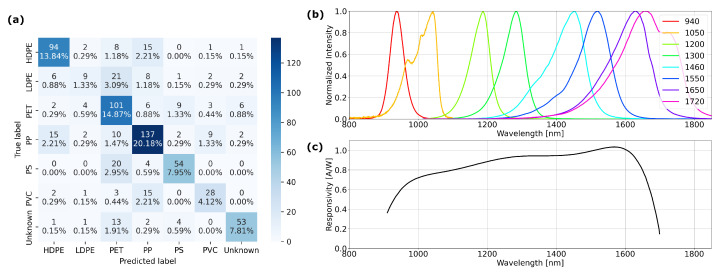
Plastic Scanner (PScanner) accuracy with (**a**) the confusion matrix showing misclassification of LDPE, PS and PVC affecting the overall accuracy, (**b**,**c**) the emission spectra of the PScanner LEDs and the sensitivity of the InGaAs detector, respectively. LED spectra were measured separately and the detector responsivity is from the manufacturer’s specifications. Misclassification is due to a lack of spectral information around 1080–1160 nm, 1320–1380 nm and the broad spectral emission and overlap above 1580 nm, combined with low responsivity of the detector.

In Figure 3, we display the confusion matrix of the best-performing algorithm (SVM) for plastic-type classification with the PlasticScanner indicating an accuracy of (0.70 ± 0.03). Although misclassifications occur for all plastic types, there is some noteworthy information in the confusion matrix. In particular, the algorithm has difficulties with LDPE as only 9 of the 49 measurements are classified correctly. Of the 40 misclassifications, 21 measurements are wrongly classified as PET. Additionally, we observe that 20 measurements of PS samples (26%) are misclassified as PET and 15 measurements of the PVC samples (31%) are wrongly classified as PP.

LDPE is often misclassified as PET, since the spectral difference (Figure 2a,b) is derived most strongly from the higher wavelengths, whereas the PScanner has LEDs no higher than 1650 and 1720 nm. Moreover, these LEDs show a strong overlap in emission spectra (see Figure 3b) and the responsivity of the InGaAs detector is low in this spectral region (see Figure 3c). Also, differentiation of plastic types from the earlier absorption lines around 1100 nm and 1350 nm (see Figure 2a,b) is difficult using the PScanner as there are gaps between the emission spectra of the LEDs, as depicted in Figure 3b. Misclassification of the PET and PVC samples by the PScanner can probably be explained along the same line of reasoning, reflecting the device’s limitations.

The SP can not use differential reflectance spectrum features and has a limited spectral resolution though a more continuous range of responsivity. Characterization of the device [9] shows response peaks with a Full Width at Half Maximum (FWHM) typically of 50 nm and 2–3 such peaks per channel (the x-axis in Figure 2d). The responsivity for all channels ranges over 850 to 1600 nm. This leads to better spectral resolution and higher responsivity at higher wavelengths, as compared to the PScanner. This technical difference reflects the accuracy obtained when applying ML models.

**Figure 4:**
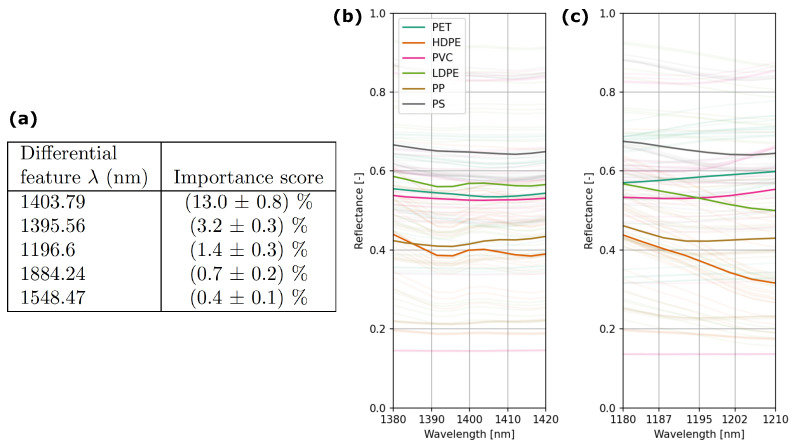
(**a**) Feature importance scores for the 5 most important features attributed to the Random Forest model approach on the NIRS data. This shows that the difference (numerical derivative) at certain wavelengths is most important in the classification. (**b**,**c**) Show the zoom of the average reflectance spectrum for the different plastic types around the three most important features used in the Random Forest model.

In Figure 4a, we display the feature importance scores of the best-performing algorithm (Random Forest with hyperparameter values for max_depth and n_estimators equal to 10 and 200, respectively) for plastic-type classification with the NIRS. Note that the feature importance scores are relatively small as the second most important feature already gives an average drop in classification accuracy of only 3.2% after randomizing its values. The low importance scores for the top features show that a combination of many different spectral values are important for the high accuracy obtained with NIRS. Moreover, we observe that the top two features characterize the change in reflectance around 1400 nm and the third feature relates to 1196.6 nm.

As can be seen in Figure 4b, different slopes for the two most important features are visible in the reflectance spectra for the different plastic types. Also at 1196.6 nm (see Figure 4c), differences in the slopes are apparent for the different plastic types. However, these slopes can only be quantified with high-end spectrometry with a high spectral resolution. The PScanner and SP approach both only use a direct reflectance measurement and both have a wide spectral response, as is apparent from the broad responsivity peaks for the SP and the broad LED emission peaks for the PScanner.

By comparing two different handheld devices for plastic detection to a high-spectral-resolution spectrometer on the same set of plastic samples, we can learn valuable lessons. First of all, a true low-cost approach comes at a price, a differential approach is not possible and a high spectral resolution cannot be obtained. This results in a lower plastic identification accuracy with the handheld devices. However, a low-cost, handheld spectroscopic device still results in reasonable plastic classification accuracy and could be beneficial in type identification.

To further expand on this idea, we propose a new avenue, which is to combine the SP and PScanner approach. By illuminating with different broad LED bands and measuring all 16 pixels of an SP sensor, one would essentially multiply the spectral bands. With 8 LEDs and 16 pixels, this would mean 128 spectral bands over the wavelength range of 850–1600 nm. And to compensate for the emission gaps in LED spectra, one could, of course, additionally mount a halogen light source for added spectral information, adding up to 144 channels. We are convinced that use of such an integrated photonics device with differential illumination would greatly improve the classification accuracy in handheld NIR sensing.

## 4. Conclusions

In this work, we have demonstrated the feasability of using handheld NIR devices for plastic-type identification as compared to a high-end NIRS, using real-world plastic samples. Even though the spectral data are varied, use of ML can obtain high accuracy when classifying different plastic types. Ideally, differential features would be used with high-resolution spectral data, but with handheld devices, this cannot be obtained. NIR spectroscopy classification using a Random Forest model showed a peak accuracy of (0.974 ± 0.005).

The handheld quantitative reflectance measurements with the SP using an SVM model gave the highest accuracy at (0.93 ± 0.01). Broad spectral responsivity with multiple peaks per pixel limit the detection accuracy. The PScanner with differential LED illumination also shows maximum accuracy with SVM at (0.70 ± 0.03). Lower accuracy is explained by gaps in spectral emission and low detector responsivity at high wavelengths. We propose a new approach using two different LEDs—a broadband lightsource and an SP detector that is sensitive to different bands to increase the spectral resolution for handheld NIR sensing.

## Figures and Tables

**Table 1:** Number of measurements for all three devices (NIR Spectrometer, SpectraPod and Plastic Scanner) divided among plastic types with an additional unknown class where the NIRS showed a spectrum lacking the normally clear spectral absorbance features of the plastic types.

Class	NIRS	SP	PScanner
PP	229	244	177
PET	158	168	131
HDPE	156	185	121
LDPE	56	71	49
PS	84	94	78
PVC	52	66	49
Unknown	78	86	74
Total	813	914	679

**Table 2:** Classification accuracy (highest value for each approach in bold) of the analyzed machine learning models applied to the results obtained with the NIR Spectrometer (NIRS), SpectraPod (SP) and the Plastic Scanner (PScanner); the highest accuracies are highlighted.

Model	NIRS	SP	PScanner
SVM	0.969 ± 0.006	**0.93 ± 0.01**	**0.70 ± 0.03**
XGBoost	0.962 ± 0.006	0.90 ± 0.03	0.69 ± 0.04
Random Forest	**0.974 ± 0.005**	0.87 ± 0.03	0.69 ± 0.03
Gaussian Naïve Bayes	0.909 ± 0.026	0.50 ± 0.04	0.38 ± 0.04

## Data Availability

The data and code are openly available in a repository via http://hhs.data.surfsara.nl/index.php/s/ageAyqwcmRZshLn (accessed on 8 May 2025) with password: THUASPhotonics!2025.

## Abbreviations

The following abbreviations are used in this manuscript:

NIRS	Near-Infrared Spectrometer
PScanner	Plastic Scanner
SP	SpectraPod
LED	Light-Emitting Diode
SVM	Support Vector Machine
XGBoost	eXtreme Gradient Boosting
PET	Polyethylene Terephthalate
HDPE	High-Density Polyethylene
PVC	Polyvinyl Chloride
LDPE	Low-Density Polyethylene
PP	Polypropylene
PS	Polystyrene
InGaAs	Indium Gallium Arsenide
